# Types of Epiglottic Collapse in Breathing Sleep Disorder and Their Impact in Clinical Practice

**DOI:** 10.1055/s-0043-1776719

**Published:** 2024-01-24

**Authors:** Elvie Zulka Kautzia Rachmawati, Susyana Tamin, Fauziah Fardizza, Rahmanofa Yunizaf, Fikri Mirza Putranto, Niken Ageng Rizki, Retno S. Wardani

**Affiliations:** 1 Department of Otorhinolaryngology-Head and Neck Surgery, Fakultas Kedokteran Universitas Indonesia, Cipto Mangunkusumo Hospital, Jakarta, Indonesia; 2 Department of Otorhinolaryngology-Head and Neck Surgery, Fakultas Kedokteran Universitas Indonesia, Universitas Indonesia Hospital, Depok, Indonesia

**Keywords:** drug-induced sleep endoscopy, epiglottic collapse, laryngopharyngeal reflux, lingual tonsil hypertrophy, obstructive sleep apnea, VOTE classification

## Abstract

**Introduction** Obstructive sleep apnea (OSA) is a severe form of sleep-disordered breathing (SDB) that is strongly correlated with comorbidities, in which epiglottic collapse (EC) and other contributing factors are involved.

**Objectives** To evaluate the occurrence of EC in OSA patients through drug-induced sleep endoscopy (DISE) and to determine the factors contributing to EC.

**Methods** A retrospective study of 37 adult patients using medical history. Patients were assessed for laryngopharyngeal reflux (LPR) and lingual tonsil hypertrophy (LTH) using reflux symptom index and reflux finding score (RFS); for OSA using polysomnography, and for airway collapse through DISE. An independent *t* -test was performed to evaluate risk factors, including the involvement of three other airway structures.

**Results** Most EC patients exhibited trap door epiglottic collapse (TDEC) (56.8%) or pushed epiglottic collapse (PEC) (29.7%). Lingual tonsil hypertrophy, RFS, and respiratory effort-related arousal (RERA) were associated with epiglottic subtypes. Laryngopharyngeal reflux patients confirmed by RFS (t(25) = −1.32, *p* = 0.197) tended to suffer PEC; LTH was significantly associated (X2(1) = 2.5, *p* = 0.012) with PEC (odds ratio [OR] value = 44) in grades II and III LTH patients; 11 of 16 TDEC patients had grade I LTH. Pushed epiglottic collapse was more prevalent among multilevel airway obstruction patients. A single additional collapse site was found only in TDEC patients.

**Conclusion** Laryngopharyngeal reflux causes repetitive acid stress toward lingual tonsils causing LTH, resulting in PEC with grade II or III LTH. Trap door epiglottic collapse requires one additional structural collapse, while at least two additional collapse sites were necessary to develop PEC. Respiratory effort-related arousal values may indicate EC.

## Introduction

Sleep-disordered breathing (SDB) is a spectrum of a disease characterized by breathing disturbances while sleeping, such as primary snoring, obstructive sleep apnea/hypopnea syndrome, upper airway resistance syndrome (UARS), Cheyne-Stokes breathing, central sleep apnea, and obstructive sleep apnea (OSA), 1 with the latter comprising 5 to 10% of cases. 2 It is caused by repetitive upper airway (UA) collapse during sleep, resulting in partial or complete obstruction of airflow. 3

At the end of SDB spectrum, OSA may be related with comorbidities such as laryngopharyngeal reflux (LPR), lingual tonsil hypertrophy (LTH), obesity, hypertension, and cardiovascular and metabolic events also frequently present with OSA. 4 5 Almost 1 billion out of 7.3 billion people in the world between the ages of 30 and 69 have OSA. 4 The prevalence of mild OSA in Asia ranges widely, from 7.8 to 77.2%.

The relationship between OSA and LPR has been reported in several studies, which has led to a bidirectional relationship theory. Increased negative thoracic pressure in OSA patients is thought to facilitate transient lower esophageal sphincter relaxation, leading to gastroesophageal reflux (GERD) and LPR. 6 Nevertheless, the inflammatory response in the pharynx due to gastric juice causes a delayed response in the pharyngeal dilator muscles and increases the risk of OSA. 6 7 Association between OSA and LTH in adults is still contentious.

Obstructive sleep apnea is commonly diagnosed through polysomnography (PSG) with apnea-hypopnea index (AHI)/respiratory disturbance index (RDI) > 5. Meanwhile, AHI/RDI scores < 5 are used to diagnose UARS, which is commonly detected through PSG and requires further evaluation using measures of respiratory effort-related arousal (RERA).

The management of OSA requires precision medicine. 8 9 To achieve such precision, airway collapse could be examined with a technique called drug-induced sleep endoscopy (DISE). Introduced by Kezirian et al., the velum, oropharynx, tongue base, epiglottis (VOTE) classification was used to evaluate the structures most commonly involved in airway narrowing, as well as the degree and configuration of obstruction. It has become the most widely used simplified assessment in DISE. 9 10

Rizki et al. 11 showed the prevalence of epiglottic collapse (EC) was 15.1%, including partial and total obstruction, laterolateral, and anteroposterior configuration. Multilevel obstructions occur more frequently (68.2%) than isolated structural collapse, with high involvement of the oropharyngeal and palatal sites. 12 13 Out of the four structures, the epiglottis is the least-recognized factor in SDB. Few studies have examined the topic of EC, although EC is a well-known phenomenon that is highly associated with the failure of conventional treatments such as dental devices, continuous positive airway pressure therapy, and upper airway surgery. 10 14 This is in accordance with a study by Abdel-Aziz et al. which stated that EC and tongue-based enlargement/LTH play a role in persistent OSA after adenotonsilectomy. 15 16 The exact mechanism of EC varies widely and remains unresolved. Several theories have proposed that EC is associated with LPR, LTH, and nasal obstruction, raising the question of whether different mechanisms must be examined to further classify EC. Therefore, in this study, we aim to evaluate the occurrence of each type of EC and its contributing factors.

## Materials and Methods

### Study Design and Population

A cross-sectional study using secondary data was performed in Cipto Mangunkusumo Hospital, Jakarta, Indonesia to evaluate the occurrence and factors contributing to EC in OSA patients. This study was approved by the Faculty of Medicine Universitas Indonesia ethical committee (0840/UN2/F1/ETIK/2018) in August 2018. The medical history of adult patients diagnosed with SDB (UARS and OSA) from January 2017 to July 2018 was evaluated with a minimum sample number of 37 based on the study calculation. Data of LPR as risk factors through reflux symptom index (RSI) questionnaires, RFS with flexible nasopharyngoscopy, body mass index (BMI), as well as polysomnography (PSG), and upper airway (UA) collapse using DISE data recording were analyzed. Patients without complete PSG and DISE recording data were excluded.

The reflux symptom index is a 9-point questionnaire that ranks symptoms from 0 (no problem) to 5 (severe problem), while the reflux finding score is an 8-point visual-based scoring system evaluated through flexible nasopharyngoscopy. A score of RSI > 13 and RFS > 7 suggests LPR. Physical examinations were also done to evaluate BMI. A BMI ≥ 25 kg/m ^2^ indicates obesity based on the Asia-Pacific BMI classification. 17

### Polysomnography

Polysomnography was done using level-2 SOMNOtouch RESP. Respiratory disturbance index (RDI) is the combination of apnea/hypopnea index (AHI) and respiratory effort-related arousal (RERA)/flow limitation index (FLI) scores. Central apnea and mixed apnea were not included in this study.

It was used to categorize OSA as mild (5–15), moderate (> 15–30), or severe (> 30). Minimum O _2_ saturation (LSatO _2_ ) or nadir SpO2 is defined as the lowest oxygen saturation during a sleep study, which was also evaluated through PSG.

Upper airway resistance syndrome (UARS) is an airflow limitation due to increased respiratory effort leading to arousal from sleep without significant desaturation (RERA). Respiratory effort-related arousal (RERA) is an event characterized by an increased respiratory response of 10 seconds or longer leading to an arousal from sleep but one that does not fulfill the criteria of hypopnea or apnea. Respiratory disturbance index score lower than 5.

Lingual tonsil hypertrophy is defined as an obstruction of the vallecula view due to the lingual tonsil, and its severity is classified into three grades (mild, moderate, and severe). Mild LTH: the lingual tonsil is prominent without obscuring the vallecula; moderate LTH: the lingual tonsil obscures the vallecula and has contact with the epiglottis; severe LTH: the vallecula is filled by the lingual tonsil, and the epiglottis is partially or fully obscured.

### Drug-induced Sleep Endoscopy

Drug-induced sleep endoscopy was performed by an otorhinolaryngologist and sedation by an anesthesiologist; a bolus of 1 mg/kg of propofol was administered until the snoring apnea cycles began and no response was exhibited by the patient following calling and tactile stimulus (Ramsay 6). 17 18 The evaluation was performed by two otorhinolaryngologists (EZKR and ST) using flexible nasopharyngoscopy (Olympus Visera 0TV-S7 video scope, light source Maxenon Xi300). Interrater variability was excellent (k > 0.81) to evaluate configuration and degree of obstruction according to the VOTE classification. Based on the configuration, airway collapse was defined as anteroposterior (AP), lateral (L), or concentric (C; a combination of AP and L). The VOTE classification defines 3 categories for the degree of obstruction: (1) no obstruction (< 25%), (2) partial obstruction (≥ 25–75%), and (3) near-total or total obstruction (> 75–100%). In this study, EC was further classified into four types: trap door (TDEC), pushed (PEC), laterolateral (LLEC), and mixed trap door and pushed (MEC).

Epiglottic collapse is a collapse of the epiglottis only, away from the tongue base. Trap door epiglottic collapse is a floppy epiglottis that prolapses into the posterior pharyngeal wall during deep inspiration. Whereas pushed epiglottis (PEC) is a condition in which the bulky tongue base pushes the epiglottis backward toward the posterior pharyngeal wall or is interpreted as a secondary collapse. Laterolateral epiglottic collapse (LLEC) is defined as an epiglottic prolapse that moves in a latero-medial direction.

### Statistical Analysis

To quantify the minimum number of samples with a confidence interval of 95%, the quantification of the sample size formula for two groups was used. After power analysis, the number of samples used was 37 participants.

Statistical analysis was performed using the IBM SPSS Statistics for Windows, Version 22.0 software (IBM Corp., Armonk, NY, USA). Data were compared using independent *t* -tests to determine the relationship between RFS and EC subtypes, chi-square tests for the relationship between gender to EC subtypes, and Mann-Whitney tests for the relationship between LTH and EC subtypes. A value of *p* < 0.05 was statistically significant.

## Results

Out of 37 patients, 21 (56.8%) had TDEC. Meanwhile, PEC was found in 29.7% of patients. However, only 3 and 2 patients had LLEC and MEC, respectively (8.1% and 5.4%). The relatively low number of LLEC and MEC patients resulted in the exclusion of these two groups from further analysis.

Demographic data are presented in Table 1 . There was no significant association between age, BMI, or gender and the occurrence of EC subtypes. However, TDEC has a higher tendency to develop in males, whereas PEC is more likely to develop in females.

**Table 1:** Risk factors for epiglottic collapse

Factors	TDEC	PEC	*p* -value
Age *	51.24 ± 3.14	47.64 ± 4.82	0.522 a
BMI *	25.97 ± 1.10	23.99 ± 1.68	0.441 a
**Gender**			0.108 b
** Male**	12 (57)	3 (27)	
** Female**	9 (43)	8 (73)	
**LTH**			** 0.012 c**
** Grade I**	14 (67)	0 (0)	
** Grade II, III**	7 (33)	11 (100)	

Abbreviations: BMI, body mass index; LPR, laryngopharyngeal reflux; LTH, lingual tonsil hypertrophy; PEC, pushed epiglottic collapse; PSG, polysomnography; RDI, respiratory disturbance index; RFS, reflux finding score; RSI, reflux symptom index; TDEC, trap door epiglottic collapse.

* Mean ± SD.

a Independent *t* -test.

b Chi-square test.

c Mann-Whitney test.

There was no statistically significant result in LPR status between TDEC and PEC, shown by RSI and RFS (12.00, 0.00–30.00 vs 15.00, 8.00–32.00; *p* = 0.401) and (7.94 ± 0.96 vs 10.00 ± 0.96; *p* = 0.197), respectively. A similar result was found regarding PSG parameters between TDEC and PEC, as can be seen from RDI and Min O _2_ Sat (5.70, 0.70–35.30 vs 4.10, 0.30–15.40; *p* = 0.416) and (90.00, 66.00–94.00 vs 93.00, 76.00–95.00; *p* = 0.619).

**Fig. 1:**
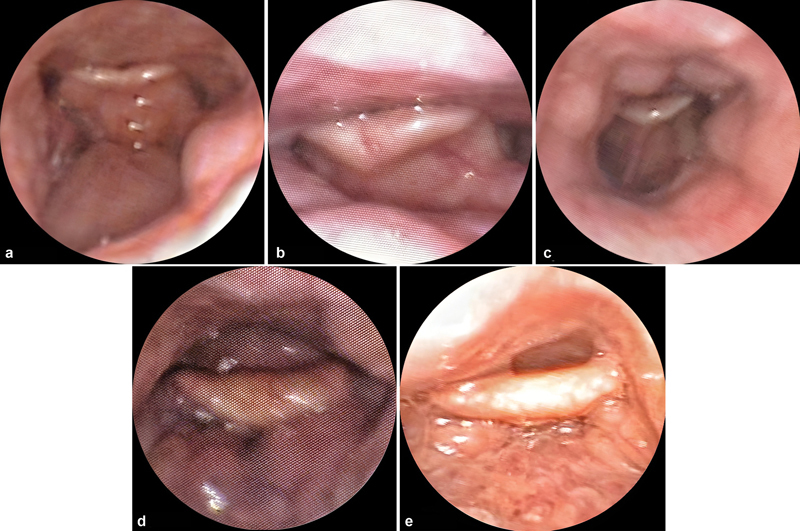
Trap door epiglottic collapse with lingual tonsil hypertrophy grades I(a), II(b), and III(c) and pushed epiglottic collapse with lingual tonsil grades II(d) and III(e).

Lingual tonsil hypertrophy, on the other hand, was significantly associated with PEC (X ^2^ (1) = 2.5, *p* = 0.012). We observed that 100% of patients with PEC had either grade II or III LTH, with an OR value of 44 (95% confidence interval [CI] 2.29–862.88) for the development of PEC. The characteristics of TDEC and PEC in relation to LTH can be seen in Fig. 1 .

In examining LPR status, neither RSI nor RFS was significantly associated with EC ( *p* = 0.401 and *p* = 0.197). The median RSI for TDEC and PEC was 12 and 15, respectively (shown in Table 1 ). Meanwhile, the mean RFS for TDEC and PEC was 7.94 and 10.00, respectively. However, LPR diagnosed through RFS showed a higher tendency to develop PEC.

In terms of PSG parameters, RDI ( *p* = 0.416) and maximum oxygen saturation (LSatO _2_ ) ( *p* = 0.619) were not significantly different between the EC types. However, patients with TDEC tended to have a higher RDI and lower LSatO _2_ than those with PEC. The occurrence of non *-* zero RERA/FLI scores for EC subtypes and LTH is presented in Table 2 .

**Table 2:** Respiratory effort-related arousal/flow limitation index scores of patients in relation to epiglottic collapse and lingual tonsil hypertrophy

RERA/FLI occurrence and type of LTH	TDEC ( *n* = 21)	PEC ( *n* = 11)
**Yes**		
** LTH grade I**	11	0
** LTH grades II and III**	5	6
**No**		
** LTH grade I**	3	0
** LTH grades II and III**	2	5

Abbreviations: FLI, flow limitation index; LTH, lingual tonsil hypertrophy; PEC, pushed epiglottic collapse; RERA, respiratory effort-related arousal; TDEC, trap door epiglottic collapse.

Twenty-two out of 32 patients had a RERA/FLI score in addition to their AHI score (shown in Table 2 ). Of these 22 patients, 16 had TDEC while 6 had PEC. In further classification, 11 of 16 TDEC patients with a RERA/FLI value had grade I LTH. Meanwhile, similar numbers of patients with and without RERA/FLI values were found among the PEC subtypes.

**Fig. 2:**
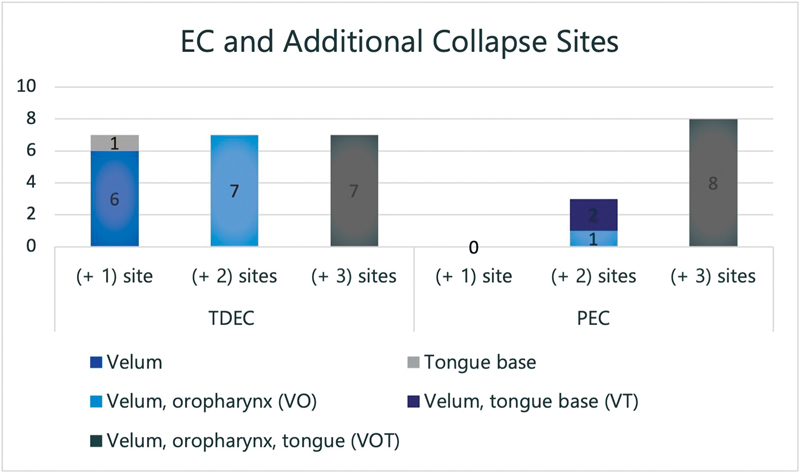
Types of epiglottic collapse in relation to velum, oropharynx, and tongue base collapse sites.

Apart from EC, other types of airway collapse (V, O, T) were also evaluated through DISE. To develop TDEC, only one additional structural collapse site was needed (shown in Fig. 2 ). In this case, 6 out of 7 TDEC patients exhibited velum collapse; of these, 67% had partial and 33% had a complete collapse. While the other patient with TDEC was presented with an additional collapse site in the tongue base. On the contrary, PEC did not exhibit a single additional collapse site.

Trap door epiglottic collapse also occurred in combination with two additional collapse sites; all these patients exhibited a combination of velum and oropharynx. Also, the collapse of at least two additional structures was needed for PEC. Two of the three PEC patients had additional collapse sites in the velum and tongue base, while the remaining individual had collapsed in the velum and oropharynx.

Out of all PEC and TDEC patients, individuals with all 4 collapse sites comprised 8 out of 11 PEC patients (72.7%) and 7 out of 21 TDEC patients (3%). This might indicate that PEC was more commonly associated with multilevel airway collapse than TDEC, especially with velum and tongue base collapse.

We calculated the prevalence of EC in the varying severities of OSA, the result is shown in Table 3 . Trap door epiglottis collapse was observed in all the OSA spectrum. In severe OSA, TDEC was also identified in two patients. Both have moderate AHI and high RERA/FLI score, (22.8/20.1) and (17.6/17.7).

**Table 3:** Obstructive sleep apnea severity based on respiratory disturbance index and epiglottic collapse configuration

OSA severity	Epiglottic collapse configuration	
	TDEC	PEC
**UARS**	9	6
**Mild**	6	3
**Moderate**	4	1
**Severe**	2	1
	21	11

Abbreviations: OSA, obstructive sleep apnea; PEC, pushed epiglottic collapse; TDEC, trap door epiglottic collapse; UARS, upper airway resistance syndrome.

## Discussion

To our knowledge, this is the first study looking at types of EC and their relationship with LTH by using DISE. In this study, the EC was classified into four types: TDEC, PEC, LLEC, and MEC. Due to the scarcity of LLEC and MEC cases, we were focusing on TDEC and PEC. 19 The remaining categories were similar to the ones used by Lin et al., 20 who further classified EC as either passive (due to posterior displacement of the base of the tongue) or active (continued isolated EC, sometimes referred to as trap door phenomenon). 21

In our study, the prevalence of TDEC and PEC was 56.8% and 29.7%, respectively. However, one previous study found opposite results, with 41.3% TDEC and 58.7% PEC. 20 Prevalence may vary due to the heterogeneity of collapse degree classification used in each study. 3 22 The study by Lin et al. 20 only included patients with severe obstruction (≥ 75%), while our study used both partial and complete obstruction (≥ 25%) as inclusion criteria. The opposite pattern of TDEC and PEC prevalence, moreover, might be influenced by the higher body weight found in Lin et al.'s study group, which had a mean BMI of 32.9 ± 7.0 compared with our mean of 25.83 ± 5.44. Body mass index (BMI) is known to play a significant role in OSA. 23 Gender, grade of LTH, RFS score, and involvement of other airway collapse sites were all important factors affecting the occurrence of TDEC and PEC (as can be seen in Table 1 , Fig. 2 ). Other factors that might contribute to this difference in prevalence include the Friedmann tongue position and LTH grade severity. 24

This study showed that TDEC has a higher tendency to develop in males, as more than half of the patients with TDEC were male. Ma et al. 19 found that males had significantly longer oropharyngeal airways ( *p* = 0.017), which were more susceptible to EC due to negative airway pressure. This phenomenon has been explained by the hollow tube law or starling theory, which states that partial obstruction of the upstream airway during inspiration may cause a greater suction force downstream, producing a greater collapsing force. 25 26

Seventeen subjects in this study were female, with a mean BMI of 26.20 ± 1.66; this was higher than the value in male patients. Increased BMI is associated with increased tongue fat. While this does not necessarily imply that the lingual tonsils are large, increased tongue fat might obstruct the view of the vallecula and put pressure on the epiglottis. 27

Moreover, high BMI and fat mass influence fat distribution around the neck area and may contribute to the development of PEC. 19 27 Our study is in line with the aforementioned statement, as 73% of PEC patients were females.

Twelve out of 16 female patients were diagnosed with LPR through their RFS scores; of these 12 females, 7 had PEC. No significant association was found between RSI or RFS scores and EC subtypes, but the RFS scores suggest that LPR tends to develop in PEC patients. Furthermore, LTH was found to be significantly associated with PEC. It was observed that all patients with PEC had either grade II or III LTH, with an OR value of 44 indicating that patients with grade II or III LTH were 44 times more likely to develop PEC. All eight female patients with PEC had grade II or III LTH, and seven of them were diagnosed with LPR. These findings agreed with previous theories proposing a relationship between PEC, LPR, and LTH. Numerous studies have stated that LPR is strongly connected with LTH, as repetitive acid stress to the tonsils causes inflammatory response and hypertrophy. 12 19 27

Research by Tang et al. 7 12 found no significant direct correlation between LTH and OSA, while other studies 27 28 stated that LPR diagnosed through the RSI and RFS might be an important factor in LTH. A multivariate analysis by Sung et al. also found an association between LTH and LPR in OSA patients due to damage to and chronic inflammation of the lymphoid tissues caused by exposure to gastrin and pepsin. 29 However, this study showed no significant association between LTH and OSA parameters alone, which might imply that the thickness and size of the lingual tonsils do not necessarily relate to OSA severity. However, there is a possibility that other structures and processes might play a role, such as EC, which are correlated with LTH. 29

Moreover, Tamin 30 stated that microtrauma in the tonsil basal cell epithelia due to repetitive acid and pepsin exposure serves as an entry point for the infiltration of human papillomavirus (HPV), especially subtypes 6 or 11. This results in partial suppression of the early viral gene, which stimulates the proliferation of basal cells and leads to lateral expansion and hypertrophy of the cells. This was further supported by 8 weeks of proton pump inhibitors (PPI) therapy, which led to changes in LTH grading; 52.2% went from grade II down to grade I, and 44.1% went from grade III to grade II ( *p* < 0.001). 31 Other studies have also identified smoking status, age, and BMI as contributing factors to LTH in OSA patients. 12 29

Minimum oxygen saturation, or LSatO2, is also correlated with EC. 14 In congruence with this, Sung et al. 21 exhibited consistent results. However, in this study, there was no significant correlation between TDEC and PEC in terms of RDI and LSatO2. This might be due to the fact that (1) the majority of patients were diagnosed with UARS or mild OSA, and/or (2) BMI among those two groups showed insignificant differences.

In this study, we were able to diagnose EC and its types using PSG and DISE. In our study, almost all EC patients (31 out of 32) exhibited velum collapse; of these, 20 out of 31 were found to have TDEC. Of these 20 patients, 6 had bilevel velum-TDEC, 7 had tri-level velum-oropharynx-TDEC, and 7 had all 4 collapse sites. Thus, the development of TDEC requires the collapse of only one additional structure apart from the epiglottis, with this being the velum in most cases. Spinowitz et al. 32 found that the velum was the most common site of UA obstruction, followed by the base of tongue and EC, with chronic nasal congestion being the most common presenting symptom. Our study supports this theory, as we noticed a minimum of two collapse sites—mostly involving the velum—in patients with TDEC. Moreover, concerning RERA, 16 out of 21 patients with TDEC were found to have a nonzero RERA/FLI score, which represents intrathoracic pressure change in UA flow limitation commonly found in UARS. 33 Upper airway resistance syndrome is a continuum of primary snoring and preceded OSA with AHI < 5; the presence of RERA and excessive daytime sleepiness are the hallmarks of UARS. 34 35 The combination of velum-TDEC and a high RERA value with AHI < 5 might indicate UARS, which can be diagnosed through PSG and further identified by DISE, that is, mid nasal exhalation/mid expiratory palatal obstruction. 32 Midnasal exhalation causes the redundant soft palate to retroflex into the nasopharynx resulting in a sudden obstruction, similar to the description of velum collapse. 32 36 Our study suggests that examiners should evaluate RDI value in conjunction with AHI and RERA to establish the development of UARS, which mostly correlates with nasal obstruction and OSA surgical failure.

Trap door epiglottic collapse, also referred to as epiglottic entrapment by Catalfumo et al., 37 has several underlying pathophysiological theories: (1) abnormalities in epiglottic cartilage, (2) loss of neuromotor control following injury to the CNS, (3) surgical resection of suprahyoid musculature and hyoepiglottic ligament, (4) increased force during inspiration causing negative intrathoracic pressure, and (5) dysregulation of mechanoreceptors. 38 39 40 Epiglottic changes such as decreased elastin, collagen, and muscle fibers lead to the loss of the hyoepiglottic ligament pars lingual, resulting in limitations to upward movement during phonation and leading to supraglottic collapse. 41 However, this histopathological change is usually found in the elderly, while our average TDEC patient's age was in the fifth decade. Our patients had no history of neurological abnormalities, nor prior surgeries or injuries. Hence, the most reasonable explanation for TDEC was increased negative intrathoracic pressure during inspiration, following the Bernoulli theory, or hyposensitivity of mechanoreceptors located in the internal branch of the superior laryngeal nerve that detect sensory information in the mucosa of the epiglottic and aryepiglottic fold. 42 43 44 Based on Kuo et al., 45 drug-induced sleep CT (DI-SCT) can visualize and diagnose more accurately, both primary (TDEC) and secondary (PEC) EC, according to which an epiglottis with 16.6 mm in length is considered as EC.

Detection of a stimulus by the mechanoreceptors causes an involuntary efferent response, mediated through the nucleus ambiguous, to adduct the vocal folds and maintain laryngeal tone. Sensorimotor integration is responsible for laryngeal tone and function, also known as the laryngeal adductor reflex (LAR). Any disruption along the afferent or efferent pathway of the LAR might alter laryngeal function and tone, as seen in the signs and symptoms of TDEC (i.e., apnea and weak laryngeal tone). 42 Another cranial nerve associated with the vagal nerve is the nasal trigeminal nerve, which innervates the velum. Together with the superior laryngeal nerve, it helps maintain airway patency by activating the airway dilator muscles. 43 44 Gastric juice due to gastroesophageal reflux disease (GERD) or LPR causes chronic inflammation and alters the sensitivity of pharyngeal receptors, resulting in delayed dilation and leading to collapse. 7 46 Hence, disruption of sensory detection in the laryngeal mechanoreceptors might lead to abnormalities in the pharyngeal airway dilator muscles, resulting in EC. This indicates that there might be an association between TDEC and velum collapse, made evident by symptoms of nasal obstruction and shown through the RERA/FLI value.

## Study Limitation

First, we did not evaluate the nasal component further, that is, inferior nasal hypertrophy, septal deviation, turbinate hypertrophy, and valve dysfunction. Those components may change the airflow velocity and resistance, which may contribute to SDB events, particularly showed us RERA number or index. In consequence, we only obtained limited data to accurately assess the association between nasal obstruction, RERA/FLI, velum collapse, and TDEC.

Secondly, we did not apply the bispectral index (BIS) and target-controlled infusion (TCI) for precise dosage monitoring. The BIS indicates the depth of the sedation which may reflect deep and light sleep. So, it facilitates collapse configuration changes during different levels of sedation. Target-controlled infusion provides a more precise dosage for obtaining the level of sedation. We used bolus IV, which may exaggerate the depth of sedation; however, the bolus was administered by a senior anesthesiologist who was an expert in the field.

Third, due to source limitation we did not use multichannel intraluminal impedance to diagnose LPR, we used RSI and RFS as tools for diagnosing LPR, which have excellent sensitivity and specificity values. We could not confirm the temporal relationship between LPR and LTH, as well as their relationship with PEC due to limited sample size; hence, further studies are needed.

Future research is needed to evaluate the role of the nasal components in EC and examine whether factors such as gender, LPR, LTH, and multilevel UA obstruction might predict the occurrence of EC subtypes.

## Clinical Relevance

During DISE, once TDEC is identified in a supine position, the patient can be positioned in the lateral head (and trunk) position (positional therapy), and TDEC can be improved after the commencement of positional therapy. This positional therapy was introduced by Vonk et al. 22 and shows a promising alternative as a standalone treatment or as a part of a combination treatment for mandibular advancement devices or less invasive forms of upper airway surgery. 22

## Conclusion

In conclusion, the occurrence of TDEC was more prevalent than PEC in this group of patients with OSA. Three additional key points may be gleaned from this study. (1) Laryngopharyngeal reflux causes repetitive acid stress to the lingual tonsils, leading to the development of LTH, which might contribute to PEC occurrence in patients with LTH grade II or III. (2) To develop TDEC, velum is needed as the additional structural collapse, whereas a minimum of two additional structural collapses (including velum) is needed to develop PEC. (3) The presence of the RERA/FLI score may increase the possibility of TDEC.
